# Mental health among single and partnered parents in South Korea

**DOI:** 10.1371/journal.pone.0182943

**Published:** 2017-08-14

**Authors:** Kyoung Ae Kong, Hee Yeon Choi, Soo In Kim

**Affiliations:** 1 Department of Preventive Medicine, College of Medicine, Ewha Womans University, Seoul, Korea; 2 Clinical Trial Center, Ewha Womans University Medical Center, Mokdong hospital, Seoul, Korea; 3 Department of Psychiatry, College of Medicine, Ewha Womans University, Seoul, Korea; National Health Research Institutes, TAIWAN

## Abstract

**Objective:**

This study compares the mental health of single parents relative to partnered parents and assesses the contribution of the social and demographic factors to this difference, examining the gender difference in it.

**Methods:**

We analyzed 12,024 single and partnered subjects, aged 30–59 years, living with children, aged 0–19 years, drawn from the 4^th^, 5^th^, and 6^th^ Korea National Health and Nutrition Examination Survey (KNHANES) dataset in South Korea conducted from 2007–2013. Mental health was evaluated by self-reported questionnaires including depressive mood for recent two weeks, presence of suicidal ideation, and the Korean version of the Alcohol Use Disorder Identification Test. Covariates included age, physical illness, socioeconomic status (family income, recipient of national basic livelihood guarantees, educational level, house ownership, job, and residential area), family structure, and support (co-residence of another adult). Multiple logistic regression was carried out and the explained fractions of each covariate was calculated.

**Results:**

Single parents had significantly poorer mental health than their partnered counterparts, with odds ratio (OR) of 2.02 (95% confidence interval (CI) 1.56–2.63) for depressive symptoms, 1.69 (95% CI 1.27–2.25) for suicidal ideation, and 1.74 (95% CI 1.38–2.20) for any of the three mental health statuses (suspicious depression, suicidal ideation, and alcohol dependence) after controlling for the covariates. The odds of depressive symptoms (OR = 3.13, 95% CI 2.50–3.93) and suicidal ideation (OR = 2.50, 95% CI 1.97–3.17) among both single fathers and mothers were higher than partnered parents. However, the odds of alcohol dependence were 3.6 times higher among single mothers than partnered mothers (OR = 3.58, 95% CI 1.81–7.08) and were 1.4 times greater among single fathers than partnered fathers (OR = 1.35, 95% CI 0.81–2.25). Socio-economic status explained more than 50% (except for substance use disorders) of the poorer mental health in single parents. These results were more remarkable for single fathers than for single mothers except for alcohol dependence. However, physical illness, family structure, and support made only minor contributions to single parents’ mental health.

**Conclusions:**

This study demonstrates that single parents have poorer mental health than partnered parents. Although lower SES is an important factor explaining poorer mental health in single parents, there are other factors we cannot explain about their poor mental health. Therefore, we should pay proper regard to identifying other factors affecting mental health and to establishing policies to support single parents.

## Introduction

According to the Korean Statistical Information Service (KOSIS), the number of single parent families in South Korea has been increasing continuously. It is reported that single parent families rose from 1.42 million in 2006 to 1.67 million families, 9.3% of the total families, in 2011 [1]. While a lower rate than Western countries (e.g., over 25% in United States, over 20% in Belgium, Chile, Denmark, France, Ireland, New Zealand, and the United Kingdom) [2], this rate shows rapid change in South Korea. This phenomenon might be caused by changes in family structure and function in South Korea [3]. Recently, the South Korean divorce rate has sharply increased with the crude divorce rate increasing more than three-fold between 1990 and 2003 (i.e., from 1.0 to 8.7 divorces per 1,000 population) [1]. In comparison to other Organization for Economic Cooperation and Development (OECD) countries, South Korea’s 2009 crude divorce rate ranked ninth among 34 countries at 2.3, while the mean OECD crude divorce rate was 1.9 [2]. The divorce rate might be associated with economic crises. South Korea experienced a severe economic crisis in the late 1990s, and the financial chaos that began in 2007 developed into full-blown economic crises in many countries [4]. Economic crises likely increased the social exclusion of vulnerable groups including children, young people, single parent families, unemployed, minorities, migrants, and elderly [5].

Single parents, especially single mothers, were reported to have poor health, especially mental health, higher risk of early death, more feeling of weakness, and poor self-perceived health than partnered parents [5–7]. The main reported mental health problems among single mothers were anxiety disorders, major depressive episodes, manic episodes, substance disorders, suicidal ideations, and other disorders [6–9]. The greater risk of poor mental health among single mothers is often explained by low socioeconomic status (SES), social support, and stress. Some studies have reported that vulnerable SES could explain 20–50% of poor mental health in single mothers [5–7, 10]). Kim et al. reported that low SES accounted for approximately 40% of the difference in self-rated health between single and partnered South Korean mothers [3]. However, there are few studies about the mental health of single fathers, especially in Asia. Although some studies have not found a difference between single and partnered parents’ health, recent Western studies have shown poorer mental health in single than partnered fathers [5, 6, 11, 12]. Our previous study in a county of South Korea also showed a poorer quality of life, more depressive symptoms, and more stress among single fathers [13]. However, there is inconsistency in the contribution of SES and other factors, such as social support, to the poor mental health of single fathers. While Cooper et al. reported that low SES and social support did not explain the excess risk of chronic mental disorders in single fathers in Britain [12], Tobias et.al reported that low SES and the absence of a co-resident adult as a proxy for social support were the main factors explaining 30–50% of single parents’ excess risk of mood disorder, suicidal ideation, and serious mental illness in New Zealand [7]. In another study in New Zealand by Collings et al., the difference in psychiatric stress between single and partnered parents among both fathers and mothers was almost fully explained by SES and social support [5]. In our previous study in Korea, low SES was significantly associated with poorer mental health among single fathers [13]. Study results about the contribution of social support were heterogeneous according to country, parental sex, and a measure of social support. However, to the best of our knowledge, there are no Asian studies that compare the mental health of single and partnered mothers and fathers with a nationally representative dataset or that assess the contribution of social, demographic, and sex differences. Understanding the contribution of each factors to poorer mental health among single fathers and mothers is essential to provide more customized support for single parents. In particular, the knowledge of the mental health problems and the contribution of the factors among single fathers, and the differences in it from among single mothers, which has not yet been well known, would be a starting point to support single fathers.

This study aimed to compare the mental health of single and partnered mothers and fathers using the dataset from a nationally representative population-based survey and to assess the contributions of factors including socioeconomic status to differences in mental health status. Additionally, we intended to examine whether there is sex difference in the association between single parenthood and mental health or in the contributions of the factors of interest. Under the cross-sectional study design, critical was the conceptual model, which was that single parenthood might increase the risk of poor mental health status and that SES, social support, and physical health could explain some part of the difference in mental health status between single and partnered patients through their mediating or confounding effect.

## Methods

### Subjects and sampling method

This study was based on data obtained from the 4^th^, 5^th^, and 6^th^ Korea National Health and Nutrition Examination Surveys (KNHANES) conducted from 2007–2013. This annual nationwide cross-sectional survey features a complex, multi-stage clustered probability sampling design to representatively sample the civilian, non-institutionalized South Korea population [14]. In 2011, the survey response rate was approximately 80%. Appropriate written informed consent for KNHANES was obtained from all participants. Patients’ records and information were anonymous and de-identified prior to analysis. Of the 23,255 subjects aged 30–59 years who participated in the health survey, we excluded 10,780 subjects without children aged 0–19 years or whose children aged 0–19 years did not participate in this survey. We additionally excluded 493subjects who did not answer the questions on marital status (12 subjects), mental health (361 subjects), or socioeconomic status such as educational level, family income, house ownership, or recipient of national basic livelihood guarantees (128 subjects). Single parents were defined as parents living with his/her child or children under 19 years old without a spouse due to divorce, bereavement, separation, neglect, disappearance, or being unmarried. Finally, 12,024 subjects (141 single fathers, 5014 partnered fathers, 407 single mothers, and 6462 partnered mothers) were included in the analysis.

### Measurements

Outcome measures of mental health status were suspicious depression, suicidal ideation, alcohol dependence, and any of these three mental health problems. Suspicious depression was assessed with questions about a continuous depressive mood for the recent two weeks or currently having depression diagnosed by a physician. Suicidal ideation was assessed with the simple question about the presence of suicidal ideation during the recent year. Alcohol dependence was assessed with the Korean version of the Alcohol Use Disorder Identification Test (AUDIT-K), which is a 10-item self-administered questionnaire with each item scored on a scale of zero to three points. Alcohol dependence was defined as greater than 20 points in AUDIT-K. This scale was developed by the World Health Organization (WHO) [15]. Kim et al. standardized the Korean version of the Alcohol Use Disorder Identification Test [16].We also defined cases having any of three mental health problems as having poor mental status.

Variables representing physical health status, SES, and social support were considered for explaining the excess risk of single parents for poor mental health status. We used the number of reported chronic illnesses as the physical health status of the subjects. Chronic illnesses included hypertension, dyslipidemia, myocardial infarction or angina, osteoarthritis or rheumatic arthritis, asthma, diabetic mellitus, thyroid disease, cancer, chronic renal failure, chronic hepatitis, and liver cirrhosis. SES measures included educational level (middle school graduate or below, high school graduate, or college graduate or above), house ownership (no house or ownership of ≥1 house), occupational category (non-manual, service and sales workers, manual workers, or outside the workforce), and family income. Income quartile groups were based on sex- and age-specific quartiles of the monthly-equivalent household income, which were calculated as monthly household income divided by the square root of the number of family members. The first income quartile group was divided into recipients of national basic livelihood guarantees and others. We used living with another adult as a proxy for social support according to Tobias et al. and Kim et al. [3, 7]. Co-resident adult was defined as an adult over 20 years old who lived with the corresponding single or partnered subject and participated in the survey. This variable was classified into living without another adult, living with any parent, or living with another adult other than parents. Demographic and basic family structure-related variables included age, number of children (1, 2, or ≥ 3), and age of the youngest child (<7, 7–12, or 12–19 years old).

### Statistical analyses

In accordance with the complex sampling design, all analyses were performed by incorporating the sampling weight, which accounts for the unequal probabilities of selection, nonresponse, sex distribution, and age distribution of the target population. Variances of all estimates were calculated using Taylor linearization method. We describe the frequency and proportion of the socio-demographic characteristics among single and partnered fathers and mothers and compared the proportions of poor mental status according to each socio-demographic characteristic with the Chi square test. Age-adjusted prevalence of poor mental health of single and partnered fathers and mothers were estimated based on the 10-year age distribution of the Korean population in 2010.

We assessed the association between single parenthood and poor mental health by logistic regression analysis considering complex survey design (with the SAS SURVEYLOGISTIC procedure) among fathers and mothers separately, adjusting for age, number of children, and age of the youngest child. The interaction of parental sex and single parenthood was tested by models including all single and partnered fathers and mothers. Then, separately by parental sex, we assessed the contribution of physical illness, SES, and living with another adult on the excess odds of poor mental health of single parents by explained fractions (EF, %), i.e., the proportion of excess risk explained by the variables. EFs were calculated as [(OR-1)-(OR_a_-1)]/(OR-1), where OR is the odds ratio (OR) of the single parent from the basic logistic regression model adjusting for age, number of children, and age of children. OR_a_ is the OR for the logistic regression model including each corresponding potential mediating or confounding factors in addition to the variables in the basic model.

## Results

### Characteristics of the study subjects

Table 1 lists the socio-demographic characteristics of the study subjects. The analysis included 141 single fathers, 5,014 partnered fathers, 407 single mothers, and 6,462 partnered mothers. Partnered mothers were significantly younger than other groups, while single fathers were older than other groups. Single parents had a significantly lower educational level, lower income, more manual job, less house ownership, more physical illness, less children aged <19 years, and older age of the youngest child. A higher proportion of single parents, especially single fathers, were living with one or both of their parents. A higher proportion of single and partnered mothers were living with another adult other than their parents. Age, chronic physical illness, educational level, and occupation were significantly associated with suspicious depression, suicidal ideation, and alcohol dependence. Family income, house ownership, number of children, and age of the youngest child were significantly associated with suspicious depression and suicidal ideation, but not with alcohol dependence. Living with parents or other adults was associated with suspicious depression and alcohol dependence.

**Table 1:** Characteristics of partnered and single parents.

			Partnered fathers	Single fathers	Partnered mothers	Single mothers	Suspicious depression	Suicidal ideation	Alcohol dependence	Any of three
			(n = 5014)	(n = 141)	(n = 6462)	(n = 407)				
		N	(col%)	(col%)	(col%)	(col%)	(%)	(%)	(%)	(%)
Sex	Men	5155					7.8***	7.2***	12.5***	21.8
	Women	6869					14.7	13.2	1.4	21.3
Age (year)	30–39	5518	37.7	25.5	50.9	30.9	10.3***	9.4*	6.2**	19.8***
	40–49	5548	51.4	56.9	45.2	57.4	11.2	10.3	7.2	22.1
	50–59	958	10.9	17.6	3.9	11.7	16.7	13.2	9.9	27.6
Number of children	1	3638	30.9	55.1	30.7	44.8	12.5*	11.1**	6.1	21.6
	2	6860	56.3	39.8	57.1	44.9	10.5	9.3	7.2	21.0
	≥3*(3* ~*6)*	1526	12.7	5.1	12.2	10.3	11.1	11.9	8.1	23.9
Age of the youngest child (years)	<7y	4900	43.3	10.8	40.0	15.5	9.5***	9.3**	6.5	19.8**
	7-<13	3960	32.3	37.1	33.3	32.7	11.4	9.8	7.8	22.2
	13-<20	3164	24.4	52.1	26.7	51.8	13.7	11.9	6.7	23.4
Number of chronic diseases	0	9531	77.9	72.8	80.6	71.4	10.2***	9.4***	6.8*	20.3***
	1	1958	17.0	14.2	15.8	21.7	14.2	12.4	7.1	25.4
	≥2 *(2* ~*7)*	535	5.1	13.0	3.6	6.9	18.1	14.2	10.4	29.9
Education	≥College	5623	51.3	26.7	41.2	21.2	8.4***	7.7***	5.8***	17.5***
	High school	5312	39.3	41.9	50.4	56.8	12.0	11.0	7.4	22.9
	≤Middle school	1089	9.3	31.3	8.4	22.0	20.9	17.6	10.6	34.0
Income quartile	Q4 (high)	2855	22.9	7.4	23.2	6.2	7.9***	6.8***	6.5	17.0***
	Q3 (mid-high)	3091	25.5	11.2	25.6	8.1	9.4	8.0	6.5	19.6
	Q2 (mid-low)	3128	27.2	24.7	26.4	14.7	10.4	10.3	7.3	21.5
	Q1 (low) except recipient of basic living	2675	23.2	35.1	23.2	43.9	15.0	13.6	7.4	25.6
	Recipients of basic living (lowest)	275	1.2	21.5	1.6	27.1	31.5	26.5	8.2	43.2
Occupation	Non-manual	3813	43.5	21.9	20.9	18.5	8.3***	6.5***	6.9***	16.9***
	Service and sales workers	1885	15.3	13.3	16.0	30.0	12.7	12.6	8.5	25.7
	Manual	2682	36.3	46.6	12.3	26.4	9.7	9.7	11.5	23.9
	Outside the workforce	3644	5.0	18.2	50.7	25.1	15.2	13.4	2.0	22.4
House ownership	No	3945	33.0	57.3	35.2	67.6	13.8***	12.7***	7.2	24.6***
	Yes	8079	67.0	42.7	64.8	32.4	9.8	8.7	6.8	19.8
Living with other adult	no other adult	9620	80.6	50.3	79.8	66.7	10.5***	10.0	7.0**	21.0***
	one or both parents	545	6.0	35.9	2.0	15.1	17.1	12.8	10.5	28.7
	adults other than parent	1859	13.4	13.8	18.2	18.2	13.0	10.3	6.0	22.3

Chronic diseases including hypertension, dyslipidemia, stroke, myocardial infarction or angina, osteoarthritis or rheumatic arthritis, asthma, diabetes mellitus, thyroid diseases, cancers, chronic renal failure, chronic hepatitis, liver cirrhosis; living with other adult over 20 years old who participated in the survey.

Differences of the characteristics among 4 groups of partnered and single fathers and mothers were all <0.001; difference among categories of each variable,

*p<0.05,

**p<0.01,

***p<0001.

Suspicious depression: continuous depressive mood for the recent 2 weeks or currently having depression diagnosed by a physician.

Suicidal ideation: the presence of suicidal ideation during the recent year.

Alcohol dependence: AUDIT-K≥20.

### Mental health of single and partnered parents

Among the four groups, single mothers had the highest prevalence of suspicious depression and suicidal ideation, while single fathers had the highest prevalence of alcohol dependence (Table 2). Comparing single to partnered mothers, single mothers had higher prevalence of suspicious depression (34.1% vs 14.6%), suicidal ideation (26.5% vs 13.0%), alcohol dependence (3.3% vs 1.1%) and any one of the three mental health problems (42.0% vs 21.0%). Comparing single to partnered fathers, single fathers had higher rates of suspicious depression (21% vs 8.4%), suicidal ideation (16.6% vs 7.5%), alcohol dependence (16.6% vs 12.1%), and any one of these mental health problems (35.7% vs 21.8%). The prevalence of suspicious depression and suicidal ideation among single fathers was higher than those among partnered mothers.

**Table 2:** Prevalence of poor mental status among partnered and single parents.

	Partnered fathers			Single fathers			Partnered mothers			Single mothers		
	(n = 5014)			(n = 141)			(n = 6462)			(n = 407)		
	n	%	(SE)	n	%	(SE)	n	%	(SE)	n	%	(SE)
Suspicious depression	371	**8.5**	(0.5)	29	**22.2**	(4.1)	840	**15.1**	(0.6)	135	**34.7**	(2.6)
Suicidal ideation	349	**7.5**	(0.5)	23	**18.2**	(3.8)	781	**13.4**	(0.6)	108	**26.6**	(2.3)
Alcohol dependence	598	**12.5**	(0.6)	23	**16.2**	(3.5)	63	**1.3**	(0.3)	13	**3.6**	(1.1)
Any of three	1051	**22.2**	(0.7)	50	**35.5**	(4.5)	1267	**21.5**	(0.7)	167	**41.5**	(2.6)

Prevalence (%) was 10-year age-standardized with Korean population in 2010.

Differences in prevalence among 4 groups of partnered and single fathers and mothers were all <0.001.

Suspicious depression: continuous depressive mood for the recent 2 weeks or currently having depression diagnosed by a physician.

Suicidal ideation: the presence of suicidal ideation during the recent year.

Alcohol dependence: AUDIT-K≥20.

In the basic model with adjustment for parental age, number of children, and age of the youngest child, single parents had more than three-fold odds for suspicious depression than partnered parents (Table 3, OR 3.28, 95% CI 2.01–5.36 among fathers; OR 3.08, 95% CI 2.41–3.94 among mothers). Single parents also had significantly higher odds for suicidal ideation than partnered parents (OR 2.86, 95% CI 1.67–4.91 among fathers; OR 2.50, 95% CI 1.94–3.23 among mothers). There were no interactions of parenthood and parental sex on suspicious depression or suicidal ideation in the model for single and partnered mothers and fathers (p for interaction: 0.81 and 0.51, respectively). The association between alcohol dependence and single parenthood was different among fathers and mothers (p for interaction 0.07). The OR for single fathers was not significantly higher than partnered fathers (OR 1.37, 95% CI 0.81–2.32), but the OR for single mothers was 3.06-fold higher than partnered mothers (95% CI 1.50–6.26). Single parents had more than two-fold odds for having any of these three mental health problems than partnered parents (OR 2.03, 95% CI 1.36–3.03 among fathers; OR 2.67, 95% CI 2.13–3.35 among mothers).

**Table 3:** Odds ratios of poor mental status for single parents compared to partnered parents.

	Fathers	(n = 5155)		Mothers	(n = 6869)	
Adjustment	OR	(95% CI)	EF (%)	OR	(95% CI)	EF (%)
**Suspicious depression**						
age, number of children, age of the youngest child	3.28	(2.01–5.36)***	Ref	3.08	(2.41–3.94)***	Ref
+ physical illness	3.16	(1.96–5.11)***	5.4	3.03	(2.37–3.88)***	2.6
+ SES	2.01	(1.16–3.49)	55.9	2.26	(1.70–3.01)***	39.3
+ living with parent/adult	2.83	(1.69–4.76)***	19.7	2.87	(2.25–3.66)***	10.3
+ physical illness + SES	1.96	(1.12–3.43)	58.0	2.27	(1.72–3.01)***	38.9
+ physical illness + living with parent/adult	2.72	(1.65–4.48)***	24.8	2.82	(2.20–3.60)***	12.8
+ SES + living with parent/adult	1.70	(0.97–2.99)	69.2	2.05	(1.55–2.71)***	49.8
+ physical illness + SES + living with parent/adult	1.66	(0.94–2.92)	71.2	2.05	(1.56–2.71)***	49.4
**Suicidal ideation**						
age, number of children, age of the youngest child	2.86	(1.67–4.91)***	Ref	2.50	(1.94–3.23)***	Ref
+ physical illness	2.78	(1.64–4.71)***	4.4	2.46	(1.90–3.19)***	2.6
+ SES	1.71	(0.96–3.04)	62.1	1.74	(1.27–2.39)**	50.6
+ living with parent/adult	2.68	(1.51–4.75)**	9.6	2.40	(1.86–3.10)***	6.8
+ physical illness + SES	1.67	(0.93–2.99)	64.0	1.74	(1.27–2.39)**	50.5
+ physical illness + living with parent/adult	2.59	(1.49–4.51)**	14.3	2.36	(1.83–3.06)***	9.3
+ SES + living with parent/adult	1.58	(0.87–2.86)	68.8	1.63	(1.19–2.23)*	58.2
+ physical illness + SES + living with parent/adult	1.54	(0.85–2.80)	70.9	1.63	(1.19–2.23)**	58.1
**Alcohol dependence (AUDIT-K≥20)**						
age, number of children, age of the youngest child	1.37	(0.81–2.32)	Ref	3.06	(1.50–6.26)**	Ref
+ physical illness	1.35	(0.80–2.27)	5.7	3.04	(1.48–6.26)*	1.1
+ SES	1.27	(0.74–2.18)	26.6	1.51	(0.59–3.91)	75.1
+ living with parent/adult	1.38	(0.79–2.40)	-1.2	2.61	(1.22–5.59)	22.0
+ physical illness + SES	1.26	(0.74–2.16)	30.2	1.52	(0.59–3.87)	75.0
+ physical illness + living with parent/adult	1.35	(0.78–2.35)	4.8	2.59	(1.20–5.59)	23.0
+ SES + living with parent/adult	1.28	(0.73–2.24)	25.1	1.19	(0.44–3.20)	90.9
+ physical illness + SES + living with parent/adult	1.27	(0.72–2.22)	28.9	1.19	(0.45–3.16)	90.8
**Any of the three diseases**						
age, number of children, age of the youngest child	2.03	(1.36–3.03)**	Ref	2.67	(2.13–3.35)***	Ref
+ physical illness	1.98	(1.34–2.93)**	4.5	2.63	(2.10–3.31)***	2.4
+ SES	1.52	(1.00–2.30)	49.5	1.88	(1.44–2.46)***	47.3
+ living with parent/adult	1.91	(1.25–2.91)*	11.5	2.52	(2.01–3.16)***	9.0
+ physical illness + SES	1.50	(0.99–2.27)	51.6	1.88	(1.45–2.46)***	47.1
+ physical illness + living with parent/adult	1.86	(1.23–2.81)*	16.2	2.48	(1.98–3.12)***	11.3
+ SES + living with parent/adult	1.42	(0.92–2.18)	59.3	1.73	(1.32–2.25)**	56.7
+ physical illness + SES + living with parent/adult	1.39	(0.91–2.15)	61.5	1.73	(1.33–2.25)**	56.5

*p<0.05,

**p<0.01,

***p<0001

(p values were adjusted by Bonferroni method, being multiplied by four, the number of outcome variables)

EF (%): the proportion of the excess explained by the variables adjusted for, calculated as (OR_basic model_—OR_adjusted for the additional variables_)/(OR_basic model_—1), where the basic model is adjusted for age, number of children, and age of the youngest child.

P interaction of parental sex and single parenthood among models including all single and partnered fathers and mothers was 0.8068 for depression, 0.5084 for suicidal ideation, 0.0713 for alcohol dependence, and 0.2052 for any of three mental health statuses.

SES, socioeconomic status.

### Explained proportion by chronic physical illness, SES, and living with parents

Compared to the basic model with adjustment for parental age, number of children, and age of the youngest child, additional adjustment for the number of chronic physical illnesses lightly lowered all ORs of single parents for suspicious depression, suicidal ideation, and alcohol dependence by 1.1%–5.4%. With additional adjustment for SES variables, SES explained 55.9% of the excess risk of suspicious depression among single fathers and 39.3% among single mothers. For suicidal ideation, the proportion explained by SES was 62.1% among single fathers and 50.6% among single mothers. For alcohol dependence, 75.1% of the excess OR of single mothers was contributed to SES. The ORs for having any of the three poor mental health problems decreased by approximately 50% after adjustment for SES variables. The proportions explained by SES were larger among fathers than mothers, except for alcohol dependence.

Additional adjustment for living with parents or another adult explained approximately 20% of the excess risk for suspicious depression and 9.6% of suicidal ideation among single fathers. Among single mothers, approximately 20% of alcohol dependence, 10.3% of suspicious depression, and 6.8% of suicidal ideation were explained. In addition, living with parents or another adult decreased the OR of having any of the three mental health problems by approximately 10% (Table 3). Surprisingly, in those models, living with parents was significantly associated with poor mental status for suspicious depression among fathers (OR 1.76) and mothers (OR 2.00), and for suicidal ideation (OR 1.55) and alcohol dependence (OR 2.50) among mothers (Table 4).

**Table 4:** Odds ratios of poor mental status for single parents compared to partnered parents.

	Fathers	(n = 5155)	Mothers	(n = 6869)
	OR^†^	(95% CI)	OR^†^	(95% CI)
**Suspicious depression**				
Single (ref: partnered)	1.66	(0.94–2.92)	2.05	(1.56–2.71) ***
Number of chronic disease (continuous)	1.22	(1.03–1.43)	1.27	(1.12–1.43) **
Education ≤Middle school (ref: ≥College)	1.65	(1.07–2.55)	2.09	(1.54–2.83) ***
High school	1.16	(0.87–1.55)	1.30	(1.06–1.60) *
Income quartile Q3 (mid-high) (ref: High)	1.12	(0.77–1.64)	1.23	(0.95–1.59)
Q2 (mid-low)	1.24	(0.85–1.80)	1.29	(1.00–1.67)
Q1 (Low)	1.56	(1.03–2.35)	1.77	(1.37–2.30) **
Recipients of basic living (lowest)	2.91	(1.39–6.12) *	2.00	(1.26–3.17) *
Occupation Service & sales workers (ref: Non-manual)	0.97	(0.67–1.40)	1.14	(0.87–1.50)
Manual	0.76	(0.55–1.05)	0.80	(0.58–1.09)
Outside the workplace	1.12	(0.66–1.89)	1.27	(1.02–1.59)
House ownership Yes (ref: No)	1.20	(0.91–1.58)	1.21	(1.01–1.46)
Living with other adult adults other than parent (ref: no other adult)	0.95	(0.67–1.36)	1.21	(0.97–1.51)
one or both parents	1.76	(1.15–2.68) *	2.00	(1.35–2.97) **
**Suicidal ideation**				
Single (ref: partnered)	1.54	(0.85–2.80)	1.63	(1.19–2.23) **
Number of chronic disease (continuous)	1.15	(0.98–1.35)	1.17	(1.01–1.35)
Education ≤Middle school (ref: ≥College)	1.31	(0.86–1.99)	1.80	(1.31–2.47) **
High school	1.12	(0.85–1.48)	1.16	(0.94–1.43)
Income quartile Q3 (mid-high) (ref: High)	1.40	(0.92–2.11)	1.00	(0.76–1.31)
Q2 (mid-low)	1.44	(0.95–2.19)	1.38	(1.05–1.81)
Q1 (Low)	1.79	(1.16–2.76) *	1.66	(1.24–2.20) **
Recipients of basic living (lowest)	3.16	(1.45–6.9) *	1.87	(1.11–3.15)
Occupation Service & sales workers (ref: Non-manual)	1.49	(1.05–2.10)	1.54	(1.13–2.10) *
Manual	1.07	(0.78–1.47)	1.24	(0.89–1.72)
Outside the workplace	1.57	(0.95–2.62)	1.35	(1.06–1.73)
House ownership Yes (ref: No)	1.23	(0.95–1.59)	1.20	(0.99–1.46)
Living with other adult adults other than parent (ref: no other adult)	0.81	(0.54–1.19)	0.92	(0.73–1.17)
one or both parents	1.31	(0.82–2.11)	1.55	(1.04–2.31)
**Alcohol dependence**				
Single (ref: partnered)	1.27	(0.72–2.22)	1.19	(0.45–3.16)
Number of chronic disease (continuous)	1.12	(0.96–1.31)	1.03	(0.60–1.74)
Education ≤Middle school (ref: ≥College)	1.96	(1.33–2.88) **	3.56	(1.26–10.03)
High school	1.41	(1.12–1.78) *	2.32	(1.02–5.28)
Income quartile Q3 (mid-high) (ref: High)	0.96	(0.73–1.26)	0.64	(0.22–1.87)
Q2 (mid-low)	0.88	(0.66–1.16)	1.73	(0.70–4.29)
Q1 (Low)	0.81	(0.59–1.12)	2.28	(0.85–6.11)
Recipients of basic living (lowest)	0.83	(0.39–1.79)	3.16	(0.69–14.52)
Occupation Service & sales workers (ref: Non-manual)	1.40	(1.06–1.85)	5.44	(1.86–15.95) **
Manual	1.26	(0.96–1.65)	3.65	(1.12–11.89)
Outside the workplace	0.88	(0.52–1.46)	2.06	(0.73–5.86)
House ownership Yes (ref: No)	1.03	(0.84–1.27)	1.12	(0.62–2.05)
Living with other adult adults other than parent (ref: no other adult)	1.00	(0.74–1.35)	0.50	(0.20–1.30)
one or both parents	0.99	(0.67–1.46)	2.50	(0.88–7.05)
**Any of the three diseases**				
Single (ref: partnered)	1.39	(0.91–2.15)	1.73	(1.33–2.25) **
Number of chronic disease (continuous)	1.17	(1.04–1.32) *	1.20	(1.07–1.35) **
Education ≤Middle school (ref: ≥College)	1.70	(1.26–2.30) **	1.85	(1.41–2.43) ***
High school	1.30	(1.08–1.56) *	1.19	(1.00–1.41)
Income quartile Q3 (mid-high) (ref: High)	1.17	(0.93–1.47)	1.12	(0.90–1.39)
Q2 (mid-low)	1.07	(0.85–1.34)	1.31	(1.04–1.63)
Q1 (Low)	1.10	(0.85–1.42)	1.59	(1.26–2.01) **
Recipients of basic living (lowest)	1.62	(0.93–2.85)	2.13	(1.38–3.29) **
Occupation Service & sales workers (ref: Non-manual)	1.35	(1.08–1.68) *	1.56	(1.21–1.99) **
Manual	1.15	(0.93–1.43)	1.07	(0.81–1.41)
Outside the workplace	1.40	(0.99–1.99)	1.36	(1.11–1.66) *
House ownership Yes (ref: No)	1.12	(0.94–1.33)	1.19	(1.02–1.39)
Living with other adult adults other than parent (ref: no other adult)	0.93	(0.73–1.19)	1.07	(0.88–1.29)
one or both parents	1.26	(0.91–1.74)	1.80	(1.26–2.57) **

*p<0.05,

**p<0.01,

***p<0001

(p values were adjusted by Bonferroni method, being multiplied by four, the number of outcome variables)

^†^Mutually adjusted for all variables listed in the table and additionally for age, number of children, and age of the youngest child

Together chronic physical illness and SES explained a similar proportion to only SES. Full adjustment for SES, physical illness, and living with parents or other adults lowered the excess odds for suspicious depression and suicidal ideation by approximately 70% and resulted in insignificant ORs of 1.66 and 1.54 among single fathers. Among mothers, full adjustment explained 49.4% of the excess odds for suspicious depression, 58.1% for suicidal ideation, 90.8% for alcohol dependence, and 56.5% for having any of the three mental health problems. Even after full adjustment, single mothers had significantly higher odds of suspicious depression (OR 2.05) and suicidal ideation (OR 1.63) than their partnered counterparts. For alcohol dependence, the OR of single relative to partnered mothers was 1.19 and not statistically significant (Table 3).

## Discussion

This study revealed that single parents had poorer mental health than partnered parents using a representative sample from South Korea. Single fathers, as well as single mothers, experienced significantly more suspicious depression and suicidal ideation than partnered counterparts. This finding corresponded to our previous study of single fathers from a single urban community in South Korea [13]. In terms of suspicious depression and suicidal ideation, single mothers have the highest prevalence, followed by single fathers, partnered mothers, and partnered fathers. This is consistent with previous studies in South Korea and Western countries [3, 5, 6, 13]. Single mothers have been regarded as one of society’s most vulnerable groups. Several factors have been proposed to explain the higher prevalence of mental health problems among single mothers, including financial problems, unemployment, lack of social support, and the burden of caring for children [17].

The prevalence of suspicious depression and suicidal ideation among single fathers was higher than partnered fathers and partnered mothers. It is well known that men are at greater risk of suicide. Male depression can be hidden [18] or under-diagnosed by traditional measures [19, 20]. Men are less likely than women to seek help [21, 22]. Therefore, in consideration of the prevalence of suspicious depression and suicidal ideation among single fathers, we should pay more attention to single fathers’ mental health.

The prevalence of alcohol dependence was much higher among fathers (16.6% of single and 12.1% of partnered) than mothers (3.3% of single and 1.1% of partnered), but the ORs of alcohol dependence were much higher among single mothers (OR 3.06, p = 0.002) than single fathers (OR 1.37, p = 0.237). This result is consistent with a previous study that reported significantly higher scores of alcohol problems in single than partnered mothers [10, 23]. Jose et al. reported negative life events (e.g., crime victim, decrease in financial position, divorce, or reporting two or more negative life events) were positively associated with heavy drinking among men, and chronic stressors (e.g., unfavorable marital status and unfavorable employment status) were also related to heavy drinking among both men and women [24]. Yankofsky et al. suggested that alcohol is used to reduce self-awareness, perceptions of negative feedback, and negative self-evaluations [25]. Therefore, single mothers suffering from more stressors and negative social discriminations might have higher ORs than single mothers. These results suggest alcohol problems are an important mental health problem that should be observed and carefully addressed in single mothers, even though the prevalence is not very high.

To our knowledge, this is the first study in Asia to estimate the contribution of factors, including socioeconomic status, to the excess risk of mental health among single parents. We found that SES explained 55.9% of the differences of suspicious depression and 62.1% of suicidal ideation between single and partnered fathers and about 39.3% and 50.6% of the differences of those between single and partnered mothers. Additionally, SES explained 75.1% of the excess risk for alcohol dependence among single mothers. SES explained more of the excess risk of poor mental health, except for alcohol dependence, among single fathers than single mothers. Physical illness and living without other adults made only minor contributions to single parents’ mental health. Tobias et al. reported that SES explained approximately 30% of the excess risk among single parents for mood disorder, substance disorder, any disorder, and 50% for suicidal ideation [7]. Cairney et al. estimated that 40% of single parents’ excess risk could be attributed to the joint effect of stress and perceived lack of social support [26]. In a study of Korean single mothers, the proportion of the excess risk of self-reported poor health among single mothers was approximately 40%, similar to this study [3]. This considerable contribution of low SES might be attributable to economic crises in South Korea. South Korea experienced a severe economic crisis in the late 1990s [27]. Additionally, the financial chaos that began in 2007 developed into a full-blown economic crisis in many countries. Economic crisis and financial hardship have resulted in a large number of single parent families due to divorce, separation, disappearance, or suicide. Economic crisis likely increased the social exclusion of vulnerable groups, including children, young people, single parent families, unemployed, minorities, migrants, and elderly [4]. Korean society has become economically polarized since the economic crisis [28]. Therefore, we hypothesized that the difference in mental health between single and partnered parents in South Korea would have been relatively more affected by socioeconomic states.

There are few mental health studies in which both fathers and mothers were included, and the EFs by each factors were assessed separately by fathers and mothers or compared, and those results were not consistent. Cooper et al. (2005) found that single fathers did not have as low SES as single mothers, and SES did not explain the excess rates of psychiatric morbidity among single fathers. Collings et al. found that the differences between single and partnered parents’ psychological distress, measured as a continuous scale about anxiety and depressive symptoms in the preceding four weeks, was larger among mothers, but was nearly fully explained by SES among both fathers and mothers [5]. On the other hand, a Canadian study by Wade et al. showed that the excess risk of having any psychiatric disorder among single mothers was higher than that of single fathers, but the proportion explained by SES was much larger among fathers (37%) than mothers (14%), which is the most similar to the present study, where the poor mental health of single fathers was also more largely explained by SES than that of single mothers. This might also be attributable to economic crisis in South Korea. Men are at a particularly increased risk of mental health problems and death due to suicide or alcohol abuse during times of economic adversity [29–31]. Generally, women suffer from a higher risk of lower income or social exclusion than men. However, the current economic crisis has reduced the sex gap in income (i.e., increased men’s risks of lower income more than women’s) rather than reducing the latter [32]. Additionally, single fathers may receive lower levels of social support than single mothers in South Korea because most material, social, and mental support programs from government and social support group focus on single mothers, and men do not consistently report psychosocial problems and distress [33, 34]. Therefore, the effect of low SES might be larger among men than women, and the difference in mental health between single and partnered parents could be explained more among men.

Physical illness makes only a minor contribution to single parents’ excess risk for any mental disorder. This might be due to the relatively young age of our sample, which ranged from 30 to 59 years, and the small number of single parents with chronic physical illness in our sample. This finding is consistent with Tobias et al.’s study that reported that poor physical health explained approximately one-tenth of the excess risk.

Living with parents or other adults, as a proxy measure for social support, explained 10–20% of the excess mental health risk of single parents. However, on the contrary to our expectation, living with parents was positively associated with the risk of poor mental health (OR 1.55–2.50), while living with an adult other than a parent was not associated with it. In other words, a co-resident parent or adult was not an adequate proxy measure of social support. Prior explanation of the difference between single and partnered parents by social support was not consistent according to countries, parental sex, and instruments. Wade et al. assessed social support measuring tangible support, affection, positive social interaction, and emotional-informational support [6, 35]. They showed that social support moderated the difference in mental health between single and partnered fathers, but not between single and partnered mothers. In a Britain study by Cooper et al [12], perceived social support assessed from seven questions did not explain the excess rates of psychiatric morbidity in single parents, especially in fathers. Co-resident adults may also have heterogeneous effects according to the subject’s age, sex, and socioeconomic status, as well as the co-resident’s number, sex, age, health status, and closeness or relationship with the single parents. Tobias et. al. reported in a study of New Zealand that lack of a co-resident adult in the household explains approximately one-quarter of the contribution to the excess mental health risk of single parents [7] and emphasized that not having another household adult is nearly as important a risk factor as low SES. Kim et al. used having natural parents alive as one proxy measure of social support in a study of Korean mothers aged 20–64 years. This was associated with a low risk of poor mental health among single mothers, but accounted for only 3.18% of the difference between single and partnered mothers in poor health risk. Living with parent/other adult in our study is not different from that of Tobias et al., but from that of Kim et al. in use of co-residence or aliveness of their parents. Our finding that the social support from living with parent did not reduce the risk of poor mental health among single parents and rather played a role as a risk factor, which could be explained by some reasons within a special context in Korea: 1) housing purchase price has increased constantly, 2) lease deposit and monthly rent price increases have put great pressure on disadvantaged groups since 2009. Therefore, living with parents might reflect low SES as they cannot afford to have separate living places or they need to share any socioeconomic support from the community or society. It might also reflect the dependence on their own parents due to health problems or denote the greater need for childcare and housekeeping for single parents. Although parents gave social support and physical help to single parents and their children, it might not be enough to overcome the effect of lower SES and the heavier burden from the greater need. Living with parents was shown as a risk of poor mental health. Therefore, living with parent should be considered as a sort of low SES or other type of hardship, and social support in South Korea needs to be assessed by a more specific instrument.

The major limitations of this study are as follows. First, our study was based on simplified and self-reported questionnaires. Such questionnaires impose real limits on the objective evaluation of symptoms. Moreover, recall biases and denials must be considered. Depressed mood and suicidal ideation were evaluated by very simplified questions. The limitations of this measure are as follows. There is a possibility that participants who were assessed having depression might include participants temporarily depressive or labile by daily life stressor. In that case, our results might report more gender differences in depression and have lower diagnostic accuracy than using the diagnosis of depression based on DSM-IV. However, it has strengths. A single question about depressive mood over a relative short period (two weeks) might decrease the possibility of reverse causality from having depression to single parenthood than using the diagnosis of depression based on DSM-IV. Wittchen et al. reported that a single question to screen for major depression have a modest impact on the recognition of depression in general population [36]. Several studies also reported that they are very meaningful and world-wide screening questions for detecting those symptoms [37, 38]. Additionally, it is very brief. It helps save time for a nationwide survey [36]. Second, this study employed cross-sectional design, which precluded the ability to determine the causal relationships from single parenthood to mental health to socio-demographic factors. The mental status over a relative short period (two weeks or one year), based on population screening (not on psychiatric diagnosis), and a presumable and generally acceptable assumption that the parent without a mental disorder would have taken the responsibility for bring up children could support the pathway leading from single parenthood to having poor mental status as a main causal direction. Nevertheless, it was possible that the mental status measured was the relapse or worsening of chronic mental disorder or that the one with a mental disorder might have been left with the children. Furthermore, on the basis of the potential relationship between SES and single parenthood (both cause and result), the contribution (explained fraction) of low SES to the relationship between parenthood and mental health status might have been made by the role of both confounder and mediator. Although we can presume that the role as confounder was larger, the confounding and the mediating effects cannot be differentiated in this study with a cross-sectional design. Finally, we used living with parent/adult as a proxy measure of social support, but it was not a predictive factor of mental health. This might be caused by not considering the demographic and physical conditions of the co-resident parent/adult or by misclassification due to nonparticipation in the survey of the study subjects’ parents. Furthermore, co-residing parent/adult might denote a heavier burden of life rather than social support. Therefore, we need to better understand if living with parent/adult is a proxy of harder life condition, unlike our original perception.

However, our study has strengths. First, we used a large and nationally representative sample of both single and partnered parents. Second, to our knowledge, this is the first study to report the contribution of SES, physical illness, and living with parent/adults to differences in mental health in Asia.

## Conclusion

This study revealed that single parents have poorer mental health than partnered parents, explained mainly by low SES. Because the mental health of single parents can be recognized by screening, we suggest that the responsible community office address the SES of single parents. Although lower SES is an important explaining factor of poorer mental health in single parents, there remain other unexplained factors that affect mental health. Therefore, we should pay proper regard to finding other factors affecting mental health and to establishing policy for supporting single parents.

## Supporting information

S1 File This is the dataset of all the data used for this manuscript. (XLSX)

S2 File This is the dataset of all the data used for this manuscript. (SAV)
